# Assessment of Cardiac Autonomic Function in Women With Polycystic Ovary Syndrome Through Ewing's Battery, Heart Rate Variability Analysis, and Composite Autonomic Symptom Score-31 Scale

**DOI:** 10.7759/cureus.45580

**Published:** 2023-09-19

**Authors:** Ragini Shrivastava, Tanusha Pathak, Pranjal Shrivastava, Shweta Patel, Sunil Chouhan, Ruchi Singh, Rachna Parashar, Shweta Mishra

**Affiliations:** 1 Physiology, All India Institute of Medical Sciences, Bhopal, IND; 2 Community and Family Medicine, Bundelkhand Medical College, Sagar, IND; 3 Obstetrics and Gynecology, All India Institute of Medical Sciences, Bhopal, IND; 4 Yoga and Naturopathy, All India Institute of Medical Sciences, Bhopal, IND

**Keywords:** heart rate variability, ewing’s battery of test, compass-31, cardiac autonomic function, polycystic ovarian syndrome

## Abstract

Introduction

Polycystic ovary syndrome (PCOS) is a frequently occurring endocrine condition prevalent in women of reproductive age characterized by chronic anovulation, hyperandrogenism, insulin resistance, and a low-grade inflammatory state. Patients with PCOS are more vulnerable to developing cardiac and metabolic co-morbidities. Sympathetic overactivity is also reported in PCOS patients.

Objective

This study aimed to assess cardiac autonomic function in PCOS by Heart Rate Variability Analysis, Ewing's Test, and Composite Autonomic Symptom Scale-31 (COMPASS-31).

Methods

Thirty female PCOS patients and 30 age-matched control females were enrolled in the study. Both cases and controls were subdivided into sub-groups based on body mass index (BMI) and waist-to-hip ratio (WHR). The cardiac autonomic functions were assessed by the COMPASS-31 questionnaire, Ewing's battery tests, and short-term heart rate variability analysis.

Results

There was no significant difference in age, BMI, and WHR of both groups. Additionally, cases had a significantly higher low frequency to high frequency (LF/HF) ratio and COMPASS-31 score and also reported more derangement in Ewing's battery test, indicating cardiac autonomic dysfunction in PCOS patients.

Conclusion

Patients with PCOS are more prone to developing cardiac and metabolic co-morbidities. Early assessment of cardiac autonomic function can prevent future complications with timely interventions. Altered autonomic function in PCOS patients can be due to hyperandrogenism and insulin resistance.

## Introduction

Polycystic ovary syndrome (PCOS) is a heterogeneous condition that affects women in their reproductive age. The prevalence of this disease is around 5-10% globally, as per the Rotterdam Criteria [1]. Irregular menstrual cycle, the sequelae of hyperandrogenism, the presence of multiple cysts in the ovary along with hirsutism, acne, and male pattern alopecia and infertility are some of the common clinical manifestations of PCOS [2].

Comorbidities such as obesity, insulin resistance (IR), type 2 diabetes, etc., are closely linked with PCOS along with hypertension and impaired lipid profile. Androgenic overactivity, which is consistent with PCOS, is a key prognostic factor in the development of these cardiovascular complications [3-5].

Additionally, obesity, which is one of the major risk factors for cardiometabolic diseases, is common with PCOS and is reported in approximately half of the PCOS-affected population [6]. Both obesity and PCOS are associated with an increased risk of metabolic and cardiovascular disease (CVD). However, there is still a paucity of data evidence on whether these are independent associations [6,7].

PCOS is also associated with hyperinsulinemia, increased blood pressure (BP), dyslipidemia, metabolic syndrome, obstructive sleep apnoea, etc. All these diseases are associated with impaired cardiac autonomic function, which is also associated with a raised risk of many major global health concerns such as depression, anxiety, hypertension, diabetes, CVD, and mortality [8]. Studies have reported that women with PCOS have reduced parasympathetic (vagal) activity and increased sympathetic nervous system activity [9-10].

Heart rate variability (HRV) analysis is a reliable tool for the assessment of cardiac autonomic nerve function (CANF) [11]. HRV measures the oscillation in successive cardiac cycles as well as the oscillations between instantaneous heart rates [12]. Previous studies have demonstrated reduced HRV to predict increased cardiac mortality [13]. HRV studies in women with PCOS revealed disruption in cardiac autonomic function at rest and during the 24-hour cycle of a day [14]. It is important to note that in most of these studies, women with PCOS had many metabolic disorders, especially obesity [12-15]. Furthermore, in a study performed on non-obese women with PCOS, normal HRV was observed [16], indicating the role of metabolic abnormalities, especially obesity, in the alteration of HRV and CANF.

Impairment in autonomic function due to obesity is via decreased vagal modulation [17]. Additionally, higher adipokines in obesity also account for increased sympathetic overactivity. Endothelial dysfunction, a surrogate marker of atherosclerosis development, commonly consistent with PCOS, can also be due to obesity, IR, and increased sympathetic tone [15-19]. Researchers also observed raised systolic (SBP) and diastolic blood pressure (DBP) in PCOS women and attributed an increment to sympathetic drive, which plays a significant role in regulating BP [20].

Ewing et al. (1970) devised a series of tests, which are now considered gold standards for the assessment of autonomic dysfunctions. These tests are now being widely used in the assessment of autonomic function in various chronic diseases such as coronary artery disease (CAD) and chronic obstructive pulmonary disease (COPD) [21]. The Composite Autonomic Symptom Scale-31 (COMPASS-31) is also used for the assessment of autonomic function. This tool was developed by the Mayo Clinic in 2012 and has been utilized in the assessment of cardiac autonomic neuropathy (CAN) in various diseases such as multiple sclerosis, polyneuropathy, Parkinson's disease, etc. [22].

In this study, we analyzed autonomic function in young female patients of PCOS via HRV, Ewing's battery, and COMPASS-31 questionnaire and compared it with that of age-matched healthy control females. At present, there has been no study on the diagnostic value of the COMPASS-31 score combined with Ewing's battery and HRV analysis in people with PCOS. Therefore, this study is aimed at utilizing these methods to evaluate cardiac autonomic dysfunction in PCOS.

## Materials and methods

This study was conducted in the autonomic function and HRV laboratory of the Department of Physiology All India Institute of Medical Sciences Bhopal in Central India after obtaining ethical approval from the Institutional Human Ethics Committee of All India Institute of Medical Science Bhopal (IHEC-LOP/2021/IM0430). 

The study included 30 female participants with PCOS (according to Rotterdam Criteria) and 30 age-matched control female participants with no symptoms of PCOS. Waist circumference was measured around the abdomen to the level of the umbilicus. Hip circumference was measured at the largest circumference around the hips. Measurement of height and weight were done by a wall-mounted stadiometer and a spring balance, respectively. Waist-to-hip ratio (WHR) and body mass index (BMI) were calculated, and participants were classified as per the Asian criterion for BMI. Heart rate (HR) was measured at baseline after five minutes of rest. Systolic blood pressure (SBP) and diastolic blood pressure (DBP) were recorded at baseline, followed by five minutes of rest using a mercury sphygmomanometer. Hirsutism was measured using the modified Ferriman-Gallwey score.

Autonomic function tests were done in the follicular phase of the menstrual cycle; the assessment was done in the amenorrhoeic period to prevent the effects of ovulatory hormones. Participants of both groups were asked not to consume any caffeinated drinks or alcohol 12 hours before the study and were asked to fast for at least four hours before the assessment.

COMPASS-31 questionnaire was administered first by the investigators. The scale has five domains for the assessment of autonomic functions [22]. The total score of all domains ranges from 0 to 100. Higher scores indicated greater autonomic dysfunction. The COMPASS-31 questionnaire prior to other assessments. Administration and evaluation of COMPASS-31 were done independently, blinded to the results of other tests. HRV was analyzed for five minutes. Fast Fourier transformation of electrocardiogram (ECG) sampled at 1000 Hz rate was done for power spectral analysis. As it was a short-term recording for a five-minute frequency, domain analysis was done recording the low-frequency component (LF; 0.04-0.15 Hz), high-frequency component (HF; 0.15-0.4 Hz), LF/HF ratio, and total power (TP).

Ewing's battery tests included tests of both sympathetic and parasympathetic function.

Parasympathetic reactivity tests

Deep Breathing Test

After five minutes of rest, participants were asked to slowly inhale and exhale at six breaths/min, with ECG being recorded continuously during the deep breathing process. The expiration-to-inspiration (E:I) ratio was then calculated. The E:I ratio is the ratio of the average of maximum RR intervals while exhaling and the average of minimum RR intervals while inhaling.

Valsalva Maneuver

Participants were asked to exhale forcefully into a mouthpiece attached to a sphygmomanometer and maintain an expiratory pressure at 40 mmHg for 15 seconds. ECG from lead II was recorded for one minute during forceful exhalation and at relaxation. Valsalva ratio (VR) was calculated as the ratio of the longest RR interval in Phase IV and the shortest RR interval in Phase II.

Lying to Standing (30:15 ratio)

This test assessed Heart rate changes upon changing from supine to standing. After five minutes of rest, participants were subjected to a 60º tilt using a passive tilt table and were kept in the same position for five min. The 30:15 ratio is the ratio of the most extended RR interval on the 30th beat after standing to the shortest RR interval around the 15th beat. 

Isometric Handgrip Test

After five minutes of rest, baseline BP was recorded using a mercury sphygmomanometer. Participants were asked to hold the hand grip dynamometer with dominant hard with full force for a few seconds to obtain maximum voluntary contraction, and the maximum force exerted was recorded. After this, the participants were asked to press the dynamometer 30% of their MVC for four minutes. Subsequent BP readings were noted at one minute, two minutes, and four minutes. ΔDBP was recorded from baseline.

Cold Pressure Test

Baseline BP was recorded after a five-minute rest using a mercury sphygmomanometer. Participants were then asked to immerse the non-dominant hand in cold water (4-6ºC) for one minute. BP was measured at one minute, one and a half minutes, and at four min. ΔDBP was recorded from baseline.

Lying to Standing

This test measures both sympathetic and parasympathetic activity. For sympathetic activity, ΔSBP was recorded on lying down and at the passive tilt. BP was noted at 30 seconds, one minute, two minutes, and five minutes.

Table 1 illustrates normal values for Ewing's battery test, and Table 2 denotes the overall scoring of Ewing's battery tests.

**Table 1:** Normal values for Ewing's battery test E:I - expiration-to-inspiration ratio; DBT - deep breathing test; LTS - lying to standing; HGT - hand grip test; CPT - cold pressure test; SBP - systolic blood pressure

Test	Normal	Borderline	Abnormal
E:I (DBT)	≥1.21	1.11-1.20	≤1.10
Valsalva ratio	≥1.21	1.11-120	≤1.10
30:15 (LTS)	≥1.04	1.01-1.03	≤1.00
HGT	≥16mmHg	11-15mmHg	≤10mmHg
CPT	≥16mmHg	11-15mmHg	≤10mmHg
SBP (LTS)	≥10mmHg	11-15mmHg	≤20mmHg

**Table 2:** Overall AFT scores as per Ewing's test AFT score - Autonomic Function Test score

Diagnosis	Ewing's parasympathetic	Ewing's sympathetic
Normal	0-2	0
Early	2-4	1
Definite	>4	≥2
Severe	Parasympathetic >4 sympathetic ≥1	

## Results

**Figure 1:**
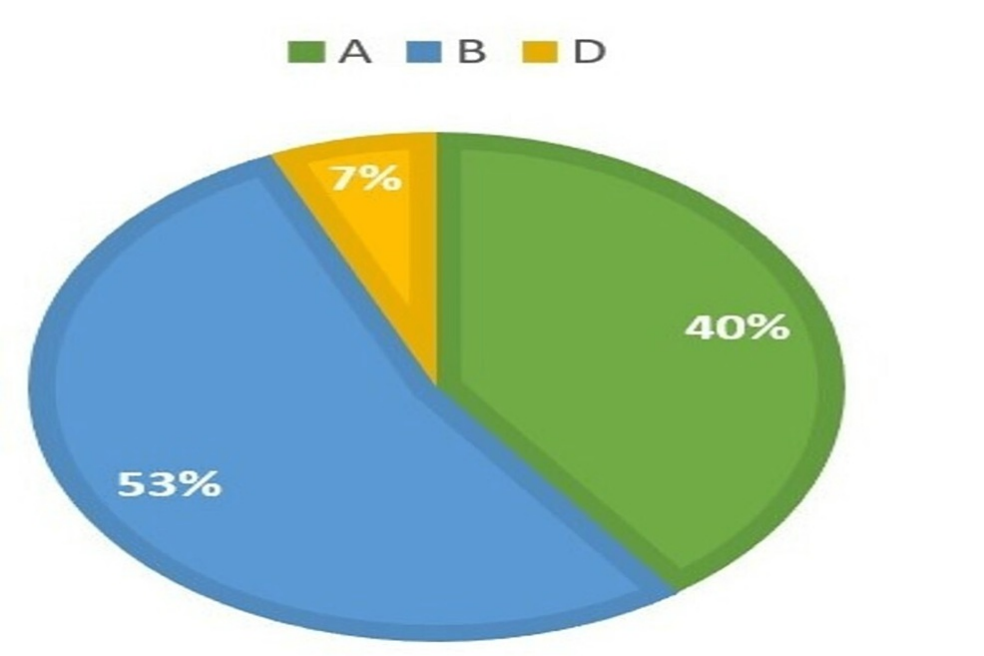
Phenotypic classification of cases

The study included 30 female participants with PCOS and 30 age-matched control participants. Phenotypic classifications of cases are illustrated in Figure 1. The cases are classified based on the Rotterdam criteria [2]. Most participants included in our study were newly diagnosed with PCOS; the mean duration of PCOS was 2.25 years, and the mean Ferriman-Gallwey score was 8.22. 

Age and anthropometric parameters (WHR and BMI) between cases and control had no significant difference as illustrated in Table 3. Slightly higher BMI and WHR were reported in patients.

**Table 3:** Classification of participants as per BMI OW - overweight; NW - normal weight; UW - under weight

Category	Age (years) (mean±SD)	WHR (mean±SD)	BMI (kg/m^2 ^)			
			BMI (mean±SD)	Obese	OW	NW
Cases (n=30)	23.16±3.367	0.83±0.106	22.19±5.396	07	4	19
Control (n=30)	24.06±4.072	0.80±0.067	20.69±3.362	02	06	22
p-value	0.362937656	0.500595	0.207482			

COMPASS-31 scores were significantly higher in cases as compared to the control population. Basal cardio-vascular parameters showed no significant difference between the two categories however, the values were slightly raised in the cases. A significant difference (p<0.05) was observed in LF/HF ratio (Table 4).

**Table 4:** Comparison of basal cardiovascular parameters, COMPASS-31 scores, HRV, and Ewing's score between cases and controls SDNN - standard deviation of NN intervals; rMSSD - root mean square of successive RR interval differences; pRR50 - percentage of successive RR intervals that differ by more than 50 ms; LF/HF - ratio of low frequency and high frequency; COMPASS-31 - Composite Autonomic Symptom Scale-31 *Significance p<0.05; **Significance p<0.005

Parameter	Case (n=30) (mean±SD)	Control (n=30) (mean±SD)	p-value
HR	73± 8.26	71.5±7.517	0.46271
SBP	112.46±7.274	111.26±7.366	0.53494
DBP	75.26±8.189	72.26±5.334	0.103742
COMPASS31	16.17±13.229	5.18±12.263	0.001808**
LF/HF Ratio	1.15±0.9	0.713±0.5	0.033162*
LF power (µ)	45.033±19.018	36.47±15.947	0.06836
HF power (µ)	52.49±17.49	60.79±13.93	0.050315
SDNN	58.09±37.58	57.76±55.88	0.978595
rMSSD	61.21±52.22	66.04±79.47	0.785387
pRR50	28.27±23.42	28.09±25.53	0.978817
Valsalva	1.62±0.35	1.6±0.24	0.823114437
30:15 Ratio	1.32±0.18	1.36±0.16	0.338084
E: I	1.19±0.17	1.21±0.21	0.68886

Table 5 denotes the scores obtained after analyzing Ewing's battery test [23]. As indicated from the table, more abnormal and borderline values in all tests are reported in cases as compared to control.

**Table 5:** Ewing's battery score E:I - expiration-to-inspiration ratio

Variables	Normal	Borderline	Abnormal
Valsalva ratio			
Control (n=30)	30	0	0
Cases (n=30)	25	2	3
Lying to standing			
Control (n=30)	30	0	0
Cases(n=30)	30	0	0
E:I ratio (deep breathing)			
Control (n=30)	28	2	0
Cases (n=30)	12	0	18
Cold pressure test			
Control (n=30)	30	0	0
Cases (n=30)	11	10	9
Hand grip test			
Control (n=30)	28	0	2
Cases (n=30)	16	6	8

## Discussion

We investigated cardiac autonomic function in patients of PCOS via HRV, Ewing's battery, and COMPASS-31 questionnaire.

Cardiac autonomic function is frequently evaluated using heart rate variability (HRV) due to its practicality and non-invasive nature. A considerably elevated LF/HF ratio was seen in the cases group as compared to age-matched controls. Saranya et al. [12] also observed comparable findings, but no significant differences were seen in other time-domain HRV parameters, such as standard deviation of NN intervals (SDNN) and root mean square of successive RR interval differences (rMSSD). However, it is worth noting that rMSSD, which reflects parasympathetic modulation of HRV, exhibited a reduction in the cases studied. The results presented here align with the findings reported by Ji et al. (2018) [24]. Yildirir et al. [25] observed a decrease in high frequency (HF) and an increase in low frequency (LF) in individuals with polycystic ovary syndrome (PCOS) compared to the control group. Our study likewise found similar results, indicating an elevated sympathetic drive and reduced vagal tone in PCOS cases. However, it is worth noting that a few studies have reported contradictory findings [26].

Previous HRV studies on PCOS have also indicated a raised sympathetic activity in PCOS patients [27], which can be associated with hormonal and metabolic characteristics that may be relevant to the pathophysiology of the syndrome. Insulin resistance (IR) and hyperandrogenism (HA) are major hormonal impairments that contribute to the pathophysiology of PCOS and may lead to sympathetic dysfunction and chronic low-grade inflammation [5]. 

Among the Ewing's battery tests, two participants reported borderline and three abnormal values in the Valsalva ratio, 18 abnormal values were reported in cases in the E:I ratio, 10 borderline, and nine abnormal values were observed in cases in the cold pressure test, and six borderline and eight abnormal values were reported in cases in hand grip test. This, again, is suggestive of autonomic dysfunction in PCOS patients, which is also like the findings of Saranya et al. [12].

This is the first study that employed the COMPASS-31 score in PCOS. We reported a significantly higher COMPASS-31 score in cases as compared to controls, which suggests an impaired autonomic function in cases. COMPASS-31 has been validated by various studies for the assessment of cardiac autonomic function. One major advantage of the COMPASS-31 is that it is simple and easy to perform in clinical practice, unlike Ewing's test and HRV, which are complicated and variable. The COMPASS-31 has a continuous range from 0 to 100, which provides a more accurate evaluation of the severity. Studies have incorporated the COMPASS-31 scale in the assessment of diabetic autonomic neuropathy (DAN), cardiac autonomic neuropathy (CAN), SFPNs, etc. [24].

An increase in sympathetic drive in PCOS is correlated with ovarian sympathetic outflow. In an animal study, rats with estrogen-induced polycystic ovaries had higher levels of norepinephrine and a high degree of transmitter release after electrical stimulation of the ovary; this may be linked to insulin resistance, hyperandrogenism, and obesity, which are commonly consistent with PCOS. Obesity plays a key role in autonomic dysregulations associated with PCOS as it leads to increased androgenic and reduced vagal modulation. Most studies have reported more alteration in autonomic function in obese PCOS patients than in normal weight [12]. However, in our studies, there was no significant difference between the BMI of both groups, and most PCOS participants were within a normal BMI range and young yet had some early indicators of altered cardiac autonomic function, which is reflected by significantly high COMPASS31 score, LF/HF ratio, and deranged Ewing's battery scores. Similarly, a study by Ribeiro et al. reported no significant fat distribution between cases and control [28]. Hyperandrogenism and Insulin resistance may contribute to increased sympathetic tone in PCOS patients rather than BMI [29]. 

Lifestyle modification can be suggested to these patients for prevention of the progression of cardiac autonomic dysfunction.

Limitations

Our study has some limitations, such as our study group being relatively small. Further larger studies are required to confirm these results. Also, no additional cardiac or biochemical tests were performed. Additionally, most participants in our study belonged to a younger age group, which may have influenced the results of the study. Studies involving women of various age groups are needed to confirm the findings.

## Conclusions

The prevalence of PCOS is increasing day by day, which is also linked to other co-morbidities such as obesity, hypertension, type II diabetes, etc., which makes PCOS patients more vulnerable to cardiac diseases as well. Early assessment of cardiac health can prevent future complications. Our study assessed cardiac autonomic function in PCOS patients via a combination of HRV analysis, Ewing's battery, and COMPASS-31 scales. We reported some early indicators of cardiac autonomic dysfunction in women with PCOS, irrespective of BMI. Further larger studies are needed to validate the use of COMPASS-31 scores for autonomic function assessment in PCOS.

## Appendices

**Table 6:** COMPASS-31 questionnaire

Question	Remark
In the past year, have you ever felt faint, dizzy, “goofy”, or had difficulty thinking soon after standing up from a sitting or lying position	1 Yes 2 No (if you marked No, please skip to question 5
2. When standing up, how frequently do you get these feelings or symptoms?	1 Rarely 2 Occasionally 3 Frequently 4 Almost Always
3. How would you rate the severity of these feelings or symptoms?	1 Mild 2 Moderate 3 Severe
4. In the past year, have these feelings or symptoms that you have experienced:	1 Gotten much worse 2 Gotten somewhat worse 3 Stayed about the same 4 Gotten somewhat better 5 Gotten much better 6 Completely gone
5. In the past year, have you ever noticed colour changes in your skin, such as red, white, or purple?	1 Yes 2 No (if you marked No, please skip to question 8)
6. What parts of your body are affected by these color changes? (Check all that apply)	1 Hands 2 Feet
7. Are these changes in your skin colour:	1 Getting much worse 2 Getting somewhat worse 3 Staying about the same 4 Getting somewhat better 5 Getting much better 6 Completely gone
8. In the past 5 years, what changes, if any, have occurred in your general body sweating?	1 I sweat much more than I used to 2 I sweat somewhat more than I used to 3 I haven’t noticed any changes in my sweating 4 I sweat somewhat less than I used to 5 I sweat much less than I used to
9. Do your eyes feel excessively dry?	1 Yes 2 No
10. Does your mouth feel excessively dry?	1 Yes 2 No
11. For the symptom of dry eyes or dry mouth that you have had for the longest period of time, is this symptom:	1 I have not had any of these symptoms 2 Getting much worse 3 Getting somewhat worse 4 Staying about the same 5 Getting somewhat better 6 Getting much better 7 Completely gone
12. In the past year, have you noticed any changes in how quickly you get full when eating a meal?	1 I get full a lot more quickly now than I used to 2 I get full more quickly now than I used to 3 I haven’t noticed any change 4 I get full less quickly now than I used to 5 I get full a lot less quickly now than I used to
13. In the past year, have you felt excessively full or persistently full (bloated feeling) after a meal	1 Never 2 Sometimes 3 A lot of the time
14. In the past year, have you vomited after a meal?	1 Never 2 Sometimes 3 A lot of the time
15. In the past year, have you had a cramping or colicky abdominal pain?	1 Never 2 Sometimes 3 A lot of the time
16. In the past year, have you had any bouts of diarrhoea?	1 Yes 2 No (if you marked No, please skip to question 20)
17. How frequently does this occur?	1 Rarely 2 Occasionally 3 Frequently 4 Constantly
18. How severe are these bouts of diarrhoea?	1 Mild 2 Moderate 3 Severe
19. Are your bouts of diarrhoea getting:	1 Much worse 2 Somewhat worse 3 Staying the same 4 Somewhat better 5 Much better 6 Completely gone
20. In the past year, have you been constipated?	1 Yes 2 No (if you marked No, please skip to question 24)
21. How frequently are you constipated?	1 Rarely 2 Occasionally 3 Frequently 4 Constantly
22. How severe are these episodes of constipation?	1 Mild times per month:____ 2 Moderate times per month:____ 3 Severe times per month:____
23. Is your constipation getting:	1 Much worse 2 Somewhat worse 3 Staying the same 4 Somewhat better 5 Much better 6 Completely gone
24. In the past year, have you ever lost control of your bladder function?	1 Never 2 Occasionally 3 Frequently 4 Constantly
25. In the past year, have you had diffic1Jlty passing urine?	1 Never 2 Occasionally 3 Frequently 4 Constantly
26. In the past year, have you had trouble completely emptying your bladder?	1 Never 2 Occasionally 3 Frequently 4 Constantly
27. In the past year, without sunglasses or tinted glasses, has bright light bothered your eyes?	1 Never {if you marked Never, please skip to question 29) 2 Occasionally 3 Frequently 4 Constantly
28. How severe is this sensitivity to bright light?	1 Mild 2 Moderate 3 Severe
29. In the past year, have you had trouble focusing your eyes?	1 Never (if you marked Never, please skip to question 31) 2 Occasionally 3 Frequently 4 Constantly
30. How severe is this focusing problem?	1 Mild 2 Moderate: times per month: 3. Severe
31, Is the most troublesome symptom with eyes (i.e. sensitivity to bright light or trouble focusing) getting	1 I have not had any of these symptoms 2 Getting much worse 3 Getting somewhat worse 4 Staying about the same 5 Getting somewhat better 6 Getting much better 7 Completely gone
